# Supplementary material for: Hearing impairment among adult foreign-born and Swedish-born individuals: A national Swedish study
Source: PLoS One. 2022 Aug 24;17(8):e0273406. doi: 10.1371/journal.pone.0273406 (PMC9401125; doi:10.1371/journal.pone.0273406)
Supplement: S1 File — (DOCX) [file pone.0273406.s001.docx]

**Supplementary information**

| **Supplementary Table 1a. Study population and number of cases in men categorized by region of origin** | | | | | | | | | | | |
| --- | --- | --- | --- | --- | --- | --- | --- | --- | --- | --- | --- |
|  | Swedish-born | | | | |  | Foreign-born | | | | |
|  | Population | |  | Events | |  | Population | |  | Events | |
|  | No. | % |  | No | % |  | No. | % |  | No | % |
| Total population | 2193544 |  |  | 108788 |  |  | 433820 |  |  | 15561 |  |
| Age (years) |  |  |  |  |  |  |  |  |  |  |  |
| 25-39 | 639144 | 29.1 |  | 13195 | 12.1 |  | 172058 | 39.7 |  | 3734 | 24.0 |
| 40-49 | 416650 | 19.0 |  | 19631 | 18.0 |  | 97270 | 22.4 |  | 3745 | 24.1 |
| 50-59 | 447600 | 20.4 |  | 34250 | 31.5 |  | 84859 | 19.6 |  | 4155 | 26.7 |
| ≥ 60 | 690150 | 31.5 |  | 41712 | 38.3 |  | 79633 | 18.4 |  | 3927 | 25.2 |
| Educational level |  |  |  |  |  |  |  |  |  |  |  |
| ≤ 9 | 711805 | 32.4 |  | 36923 | 33.9 |  | 176132 | 40.6 |  | 5059 | 32.5 |
| 10-12 | 626365 | 28.6 |  | 29569 | 27.2 |  | 85838 | 19.8 |  | 3654 | 23.5 |
| > 12 | 855374 | 39.0 |  | 42296 | 38.9 |  | 171850 | 39.6 |  | 6848 | 44.0 |
| Region of residence |  |  |  |  |  |  |  |  |  |  |  |
| Large cities | 736511 | 33.6 |  | 32952 | 30.3 |  | 127773 | 29.5 |  | 5772 | 37.1 |
| Southern Sweden | 1015466 | 46.3 |  | 54210 | 49.8 |  | 89423 | 20.6 |  | 4692 | 30.2 |
| Northern Sweden | 441567 | 20.1 |  | 21626 | 19.9 |  | 216624 | 49.9 |  | 5097 | 32.8 |
| Marital status |  |  |  |  |  |  |  |  |  |  |  |
| Married | 1248157 | 56.9 |  | 77702 | 71.4 |  | 327890 | 75.6 |  | 11846 | 76.1 |
| Not married | 945387 | 43.1 |  | 31086 | 28.6 |  | 105930 | 24.4 |  | 3715 | 23.9 |
| Neighborhood deprivation |  |  |  |  |  |  |  |  |  |  |  |
| Low | 344166 | 15.7 |  | 18803 | 17.3 |  | 22100 | 5.1 |  | 1266 | 8.1 |
| Middle | 1158004 | 52.8 |  | 59928 | 55.1 |  | 91186 | 21.0 |  | 4581 | 29.4 |
| High | 248226 | 11.3 |  | 11653 | 10.7 |  | 48517 | 11.2 |  | 2007 | 12.9 |
| Unknown | 443148 | 20.2 |  | 18404 | 16.9 |  | 272017 | 62.7 |  | 7707 | 49.5 |
| Hospital diagnosis of tinnitus | 30529 | 1.4 |  | 18663 | 17.2 |  | 5597 | 1.3 |  | 3191 | 20.5 |
| Hospital diagnosis of intracranial trauma | 62259 | 2.8 |  | 4411 | 4.1 |  | 8695 | 2.0 |  | 575 | 3.7 |
| Hospital diagnosis of brain tumor | 13439 | 0.6 |  | 1786 | 1.6 |  | 1883 | 0.4 |  | 263 | 1.7 |
| Hospital diagnosis of stroke | 232631 | 10.6 |  | 14654 | 13.5 |  | 24940 | 5.7 |  | 1657 | 10.6 |
| Hospital diagnosis of hypertension | 421114 | 19.2 |  | 33310 | 30.6 |  | 56094 | 12.9 |  | 4234 | 27.2 |
| Hospital diagnosis of CHD | 308957 | 14.1 |  | 23318 | 21.4 |  | 43800 | 10.1 |  | 3190 | 20.5 |
| Hospital diagnosis of COPD | 129977 | 5.9 |  | 8811 | 8.1 |  | 20042 | 4.6 |  | 1358 | 8.7 |
| Hospital diagnosis of cancer (except brain tumors) | 415757 | 19.0 |  | 30408 | 28.0 |  | 43376 | 10.0 |  | 2962 | 19.0 |
| Hospital diagnosis of diabetes | 188212 | 8.6 |  | 12634 | 11.6 |  | 33983 | 7.8 |  | 2256 | 14.5 |
| Hospital diagnosis of arthropathy | 427865 | 19.5 |  | 31582 | 29.0 |  | 53637 | 12.4 |  | 3856 | 24.8 |
| Hospital diagnosis of dementia | 64757 | 3.0 |  | 4157 | 3.8 |  | 6052 | 1.4 |  | 419 | 2.7 |
| Hospital diagnosis of depression | 68947 | 3.1 |  | 4480 | 4.1 |  | 17056 | 3.9 |  | 1091 | 7.0 |
| Hospital diagnosis of visual impairment | 5289 | 0.2 |  | 516 | 0.5 |  | 959 | 0.2 |  | 116 | 0.7 |

CHD: Coronary heart disease; COPD: Chronic obstructive pulmonary disease

| **Supplementary Table 1b. Study population and number of cases in women categorized by region of origin** | | | | | | | | | | | |
| --- | --- | --- | --- | --- | --- | --- | --- | --- | --- | --- | --- |
|  | Swedish born | | | | |  | Foreign born | | | | |
|  | Population | |  | Events | |  | Population | |  | Events | |
|  | No. | % |  | No | % |  | No. | % |  | No | % |
| Total population | 2409298 |  |  | 105260 |  |  | 427583 |  |  | 14562 |  |
| Age (years) |  |  |  |  |  |  |  |  |  |  |  |
| 25-39 | 665404 | 27.6 |  | 13088 | 12.4 |  | 170634 | 39.9 |  | 3361 | 23.1 |
| 40-49 | 431899 | 17.9 |  | 17830 | 16.9 |  | 95450 | 22.3 |  | 3171 | 21.8 |
| 50-59 | 461985 | 19.2 |  | 27858 | 26.5 |  | 73026 | 17.1 |  | 3285 | 22.6 |
| ≥ 60 | 850010 | 35.3 |  | 46484 | 44.2 |  | 88473 | 20.7 |  | 4745 | 32.6 |
| Educational level |  |  |  |  |  |  |  |  |  |  |  |
| ≤ 9 | 808926 | 33.6 |  | 36182 | 34.4 |  | 182471 | 42.7 |  | 5410 | 37.2 |
| 10-12 | 726862 | 30.2 |  | 34780 | 33.0 |  | 88852 | 20.8 |  | 4092 | 28.1 |
| > 12 | 873510 | 36.3 |  | 34298 | 32.6 |  | 156260 | 36.5 |  | 5060 | 34.7 |
| Region of residence |  |  |  |  |  |  |  |  |  |  |  |
| Large cities | 826001 | 34.3 |  | 35405 | 33.6 |  | 134204 | 31.4 |  | 6013 | 41.3 |
| Southern Sweden | 1117035 | 46.4 |  | 50846 | 48.3 |  | 96224 | 22.5 |  | 4477 | 30.7 |
| Northern Sweden | 466262 | 19.4 |  | 19009 | 18.1 |  | 197155 | 46.1 |  | 4072 | 28.0 |
| Marital status |  |  |  |  |  |  |  |  |  |  |  |
| Married | 2138383 | 88.8 |  | 94493 | 89.8 |  | 396715 | 92.8 |  | 13262 | 91.1 |
| Not married | 270915 | 11.2 |  | 10767 | 10.2 |  | 30868 | 7.2 |  | 1300 | 8.9 |
| Neighborhood deprivation |  |  |  |  |  |  |  |  |  |  |  |
| Low | 367637 | 15.3 |  | 17372 | 16.5 |  | 25279 | 5.9 |  | 1186 | 8.1 |
| Middle | 1288332 | 53.5 |  | 56497 | 53.7 |  | 101845 | 23.8 |  | 4741 | 32.6 |
| High | 280734 | 11.7 |  | 11397 | 10.8 |  | 48736 | 11.4 |  | 1873 | 12.9 |
| Unknown | 472595 | 19.6 |  | 19994 | 19.0 |  | 251723 | 58.9 |  | 6762 | 46.4 |
| Hospital diagnosis of tinnitus | 30433 | 1.3 |  | 15432 | 14.7 |  | 5225 | 1.2 |  | 2488 | 17.1 |
| Hospital diagnosis of intracranial trauma | 55206 | 2.3 |  | 3542 | 3.4 |  | 7869 | 1.8 |  | 533 | 3.7 |
| Hospital diagnosis of brain tumor | 18747 | 0.8 |  | 2175 | 2.1 |  | 2744 | 0.6 |  | 315 | 2.2 |
| Hospital diagnosis of stroke | 229342 | 9.5 |  | 11983 | 11.4 |  | 22966 | 5.4 |  | 1425 | 9.8 |
| Hospital diagnosis of hypertension | 463684 | 19.2 |  | 32046 | 30.4 |  | 61122 | 14.3 |  | 4338 | 29.8 |
| Hospital diagnosis of CHD | 221820 | 9.2 |  | 14496 | 13.8 |  | 28193 | 6.6 |  | 2000 | 13.7 |
| Hospital diagnosis of COPD | 165680 | 6.9 |  | 9983 | 9.5 |  | 24369 | 5.7 |  | 1509 | 10.4 |
| Hospital diagnosis of cancer (except brain tumors) | 420007 | 17.4 |  | 24588 | 23.4 |  | 44858 | 10.5 |  | 2657 | 18.2 |
| Hospital diagnosis of diabetes | 156544 | 6.5 |  | 9199 | 8.7 |  | 24735 | 5.8 |  | 1520 | 10.4 |
| Hospital diagnosis of arthropathy | 544003 | 22.6 |  | 35391 | 33.6 |  | 76214 | 17.8 |  | 4987 | 34.2 |
| Hospital diagnosis of dementia | 93607 | 3.9 |  | 4680 | 4.4 |  | 8978 | 2.1 |  | 541 | 3.7 |
| Hospital diagnosis of depression | 113224 | 4.7 |  | 6416 | 6.1 |  | 24777 | 5.8 |  | 1370 | 9.4 |
| Hospital diagnosis of visual impairment | 6800 | 0.3 |  | 623 | 0.6 |  | 1080 | 0.3 |  | 102 | 0.7 |

CHD: Coronary heart disease; COPD: Chronic obstructive pulmonary disease

| **Supplementary** **Table 2a. Background factors for hearing impairment showing Hazard Ratios (HRs) with 99% confidence interval (99% CI) in Swedish-born and foreign-born men** | | | | | | | |
| --- | --- | --- | --- | --- | --- | --- | --- |
|  | Born in Sweden | | |  | Foreign-born | | |
|  | HR* | 99% CI | |  | HR* | 99% CI | |
| Birth year | 0.98 | 0.98 | 0.98 |  | 0.99 | 0.98 | 0.99 |
| Educational level (ref. ≤ 9 years) |  |  |  |  |  |  |  |
| 10-12 | 1.10 | 1.07 | 1.12 |  | 1.21 | 1.14 | 1.30 |
| >12 | 1.10 | 1.08 | 1.12 |  | 1.31 | 1.23 | 1.38 |
| Region of residence (ref. Large cities) |  |  |  |  |  |  |  |
| Southern Sweden | 1.05 | 1.03 | 1.08 |  | 1.07 | 1.00 | 1.15 |
| Northern Sweden | 0.95 | 0.93 | 0.98 |  | 0.79 | 0.74 | 0.84 |
| Marital status (ref. Married) | 1.30 | 1.27 | 1.33 |  | 1.25 | 1.18 | 1.32 |
| Neighborhood deprivation (ref. Low) |  |  |  |  |  |  |  |
| Middle | 1.03 | 1.01 | 1.06 |  | 1.01 | 0.92 | 1.10 |
| High | 1.03 | 1.00 | 1.07 |  | 0.93 | 0.83 | 1.03 |
| Unknown | 0.81 | 0.78 | 0.84 |  | 0.91 | 0.83 | 1.00 |
| Hospital diagnosis of tinnitus | 18.77 | 18.34 | 19.22 |  | 19.95 | 18.82 | 21.14 |
| Hospital diagnosis of intracranial trauma | 1.27 | 1.22 | 1.33 |  | 1.29 | 1.14 | 1.46 |
| Hospital diagnosis of brain tumor | 2.43 | 2.27 | 2.60 |  | 2.32 | 1.94 | 2.78 |
| Hospital diagnosis of stroke | 1.06 | 1.03 | 1.09 |  | 1.13 | 1.04 | 1.22 |
| Hospital diagnosis of hypertension | 1.19 | 1.17 | 1.22 |  | 1.32 | 1.24 | 1.40 |
| Hospital diagnosis of CHD | 1.18 | 1.15 | 1.21 |  | 1.27 | 1.19 | 1.35 |
| Hospital diagnosis of COPD | 1.19 | 1.16 | 1.23 |  | 1.34 | 1.23 | 1.45 |
| Hospital diagnosis of cancer (except brain tumors) | 1.27 | 1.24 | 1.30 |  | 1.36 | 1.27 | 1.44 |
| Hospital diagnosis of diabetes | 1.09 | 1.06 | 1.12 |  | 1.25 | 1.16 | 1.33 |
| Hospital diagnosis of arthropathy | 1.30 | 1.28 | 1.33 |  | 1.42 | 1.35 | 1.50 |
| Hospital diagnosis of dementia | 1.02 | 0.98 | 1.07 |  | 1.20 | 1.04 | 1.39 |
| Hospital diagnosis of depression | 1.10 | 1.05 | 1.15 |  | 1.32 | 1.21 | 1.45 |
| Hospital diagnosis of visual impairment | 1.35 | 1.19 | 1.53 |  | 1.77 | 1.36 | 2.31 |
|  |  |  |  |  |  |  |  |

CHD: Coronary heart disease; COPD: Chronic obstructive pulmonary disease

*Fully adjusted, i.e. adjusted for age, region of residence in Sweden, educational level, and marital status, neighborhood deprivation, and comorbidities

| **Supplementary Table 2b. Background factors for hearing impairment showing Hazard Ratios (HRs) with 99% confidence interval (99% CI) in Swedish-born and foreign-born women** | | | | | | | |
| --- | --- | --- | --- | --- | --- | --- | --- |
|  | Born in Sweden | | |  | Foreign born | | |
|  | HR* | 99% CI | |  | HR* | 99% CI | |
| Birth year | 0.97 | 0.97 | 0.97 |  | 0.98 | 0.98 | 0.98 |
| Educational level (ref. ≤ 9 years) |  |  |  |  |  |  |  |
| 10-12 | 1.21 | 1.18 | 1.23 |  | 1.30 | 1.22 | 1.38 |
| >12 | 1.20 | 1.17 | 1.23 |  | 1.25 | 1.18 | 1.33 |
| Region of residence (ref. Large cities) |  |  |  |  |  |  |  |
| Southern Sweden | 1.05 | 1.02 | 1.07 |  | 1.00 | 0.94 | 1.07 |
| Northern Sweden | 0.93 | 0.91 | 0.96 |  | 0.81 | 0.76 | 0.86 |
| Marital status (ref. Married) | 1.11 | 1.07 | 1.14 |  | 1.02 | 0.94 | 1.11 |
| Neighborhood deprivation (ref. Low) |  |  |  |  |  |  |  |
| Middle | 0.98 | 0.95 | 1.00 |  | 1.03 | 0.94 | 1.13 |
| High | 0.96 | 0.93 | 0.99 |  | 0.98 | 0.88 | 1.09 |
| Unknown | 0.93 | 0.90 | 0.96 |  | 0.96 | 0.87 | 1.06 |
| Hospital diagnoses: |  |  |  |  |  |  |  |
| Tinnitus | 16.02 | 15.62 | 16.43 |  | 15.95 | 14.96 | 17.01 |
| Intracranial trauma | 1.23 | 1.17 | 1.29 |  | 1.33 | 1.17 | 1.51 |
| Brain tumor | 2.27 | 2.13 | 2.41 |  | 2.01 | 1.70 | 2.36 |
| Stroke | 1.02 | 0.99 | 1.05 |  | 1.14 | 1.05 | 1.25 |
| Hypertension | 1.19 | 1.16 | 1.21 |  | 1.31 | 1.24 | 1.39 |
| Chd | 1.14 | 1.11 | 1.17 |  | 1.21 | 1.12 | 1.30 |
| Copd | 1.22 | 1.19 | 1.26 |  | 1.25 | 1.16 | 1.35 |
| Cancer (except brain tumors) | 1.19 | 1.17 | 1.22 |  | 1.26 | 1.18 | 1.34 |
| Diabetes | 1.09 | 1.06 | 1.13 |  | 1.18 | 1.09 | 1.28 |
| Arthropathy | 1.31 | 1.29 | 1.34 |  | 1.47 | 1.39 | 1.54 |
| Dementia | 0.88 | 0.84 | 0.92 |  | 1.05 | 0.92 | 1.19 |
| Depression | 1.19 | 1.15 | 1.24 |  | 1.38 | 1.27 | 1.50 |
| Visual impairment | 1.37 | 1.22 | 1.54 |  | 1.64 | 1.24 | 2.18 |
|  |  |  |  |  |  |  |  |

CHD: Coronary heart disease; COPD: Chronic obstructive pulmonary disease

*Fully adjusted, i.e. adjusted for age, region of residence in Sweden, educational level, and marital status, neighborhood deprivation, and comorbidities

| **Supplementary Table 3. Study population and number of cases of hearing impairment in first-generation immigrants** | | | | | |
| --- | --- | --- | --- | --- | --- |
|  | Men | |  | Women | |
|  | Population | No. of events | | Population | No. of events |
| Sweden | 2193544 | 108788 |  | 2409298 | 105260 |
| Denmark | 23292 | 812 |  | 21198 | 692 |
| Finland | 88923 | 3306 |  | 118940 | 4580 |
| Iceland | 3331 | 46 |  | 3296 | 43 |
| Norway | 19910 | 581 |  | 26581 | 888 |
| France | 3269 | 73 |  | 2414 | 41 |
| Greece | 9500 | 224 |  | 6721 | 122 |
| Italy | 5829 | 187 |  | 2900 | 63 |
| Spain | 3622 | 91 |  | 2296 | 54 |
| Other Southern Europe | 2413 | 62 |  | 1672 | 31 |
| The Netherlands | 3650 | 105 |  | 2580 | 80 |
| UK and Ireland | 11385 | 200 |  | 6194 | 123 |
| Germany | 17413 | 776 |  | 18793 | 910 |
| Austria | 3502 | 146 |  | 2454 | 94 |
| Other Western Europe | 2026 | 61 |  | 1588 | 41 |
| Bosnia | 14199 | 637 |  | 12346 | 357 |
| Yugoslavia | 27429 | 1229 |  | 23522 | 859 |
| Croatia | 2091 | 94 |  | 1798 | 67 |
| Romania | 3965 | 130 |  | 3984 | 113 |
| Bulgaria | 1348 | 34 |  | 1315 | 39 |
| Other Eastern Europe | 2903 | 80 |  | 2142 | 53 |
| Estonia | 3780 | 200 |  | 4496 | 204 |
| Latvia | 1357 | 42 |  | 1552 | 37 |
| Poland | 11672 | 371 |  | 17723 | 559 |
| Other Central Europe | 2977 | 144 |  | 3120 | 131 |
| Hungary | 6295 | 248 |  | 5339 | 229 |
| Africa | 24827 | 590 |  | 15385 | 379 |
| Northern America | 9353 | 224 |  | 7924 | 172 |
| Chile | 9284 | 342 |  | 8751 | 298 |
| Southern America | 6800 | 190 |  | 7338 | 200 |
| Turkey | 13918 | 701 |  | 11198 | 521 |
| Lebanon | 7920 | 382 |  | 5576 | 214 |
| Iran | 19354 | 891 |  | 14644 | 582 |
| Iraq | 25960 | 1281 |  | 17125 | 630 |
| Other Asia | 33925 | 914 |  | 35964 | 927 |
| Russia | 3993 | 111 |  | 6604 | 177 |
| Others | 2405 | 56 |  | 2110 | 52 |
| All | 2627364 | 124349 |  | 2836881 | 119822 |

| **Supplementary Table 4a. Incidence of hearing impairment in first-generation male immigrants vs Swedish-born men expressed as hazard ratios (HR) with 99% confidence intervals (99% CI)** | | | | | | | | | | | | | |
| --- | --- | --- | --- | --- | --- | --- | --- | --- | --- | --- | --- | --- | --- |
|  |  | Model 1 | | |  | Model 2 | | |  | Model 3 | | |  |
|  | Obs. | HR | 99% CI | |  | HR | 99% CI | |  | HR | 99% CI | |  |
| Sweden | 108788 | 1 |  |  |  | 1 |  |  |  | 1 |  |  |  |
| **Nordic countries** | 4745 | **0.68** | **0.65** | **0.71** |  | **0.79** | **0.75** | **0.82** |  | **0.82** | **0.79** | **0.86** |  |
| Denmark | 812 | **0.67** | **0.61** | **0.74** |  | **0.75** | **0.68** | **0.83** |  | **0.81** | **0.73** | **0.90** |  |
| Finland | 3306 | **0.71** | **0.68** | **0.75** |  | **0.84** | **0.79** | **0.88** |  | **0.85** | **0.81** | **0.90** |  |
| Iceland | 46 | **0.33** | **0.22** | **0.51** |  | **0.41** | **0.27** | **0.62** |  | **0.47** | **0.31** | **0.71** |  |
| Norway | 581 | **0.58** | **0.51** | **0.65** |  | **0.68** | **0.60** | **0.76** |  | **0.74** | **0.66** | **0.84** |  |
| **Southern Europe** | 637 | **0.53** | **0.47** | **0.59** |  | **0.64** | **0.57** | **0.72** |  | **0.68** | **0.60** | **0.76** |  |
| France | 73 | **0.59** | **0.42** | **0.82** |  | 0.72 | 0.51 | 1.00 |  | 0.79 | 0.56 | 1.10 |  |
| Greece | 224 | **0.47** | **0.38** | **0.56** |  | **0.57** | **0.47** | **0.69** |  | **0.62** | **0.51** | **0.75** |  |
| Italy | 187 | **0.57** | **0.46** | **0.70** |  | **0.70** | **0.57** | **0.87** |  | **0.71** | **0.57** | **0.87** |  |
| Spain | 91 | **0.55** | **0.41** | **0.74** |  | **0.68** | **0.51** | **0.92** |  | **0.71** | **0.53** | **0.96** |  |
| Other Southern Europe | 62 | **0.59** | **0.41** | **0.85** |  | **0.67** | **0.47** | **0.97** |  | 0.70 | 0.49 | 1.00 |  |
| **Western Europe** | 1288 | **0.73** | **0.67** | **0.79** |  | **0.81** | **0.74** | **0.87** |  | **0.84** | **0.78** | **0.91** |  |
| The Netherlands | 105 | **0.74** | **0.56** | **0.97** |  | 0.86 | 0.65 | 1.13 |  | 0.92 | 0.70 | 1.21 |  |
| UK and Ireland | 200 | **0.46** | **0.38** | **0.56** |  | **0.55** | **0.45** | **0.68** |  | **0.61** | **0.50** | **0.75** |  |
| Germany | 776 | **0.86** | **0.78** | **0.96** |  | 0.92 | 0.83 | 1.02 |  | 0.93 | 0.84 | 1.03 |  |
| Austria | 146 | **0.73** | **0.57** | **0.92** |  | **0.78** | **0.62** | **0.99** |  | 0.79 | 0.62 | 1.00 |  |
| Other Western Europe | 61 | **0.64** | **0.45** | **0.93** |  | 0.77 | 0.53 | 1.11 |  | 0.87 | 0.61 | 1.26 |  |
| **Eastern Europe** | 2204 | 1.01 | 0.95 | 1.07 |  | **1.14** | **1.07** | **1.21** |  | **1.08** | **1.01** | **1.15** |  |
| Bosnia | 637 | **1.15** | **1.02** | **1.29** |  | **1.52** | **1.36** | **1.71** |  | **1.32** | **1.18** | **1.49** |  |
| Yugoslavia | 1229 | 0.97 | 0.90 | 1.06 |  | 1.07 | 0.99 | 1.16 |  | 1.04 | 0.96 | 1.13 |  |
| Croatia | 94 | 1.04 | 0.77 | 1.39 |  | 1.04 | 0.77 | 1.39 |  | 0.97 | 0.73 | 1.31 |  |
| Romania | 130 | 0.87 | 0.67 | 1.11 |  | 0.88 | 0.68 | 1.12 |  | 0.82 | 0.64 | 1.06 |  |
| Bulgaria | 34 | 0.65 | 0.40 | 1.06 |  | 0.74 | 0.46 | 1.21 |  | 0.81 | 0.50 | 1.33 |  |
| Other Eastern Europe | 80 | 1.06 | 0.77 | 1.46 |  | 1.16 | 0.84 | 1.60 |  | 1.21 | 0.88 | 1.67 |  |
| **Baltic countries** | 242 | 0.91 | 0.76 | 1.10 |  | 0.95 | 0.79 | 1.14 |  | 0.98 | 0.82 | 1.18 |  |
| Estonia | 200 | 0.92 | 0.75 | 1.13 |  | 0.95 | 0.78 | 1.17 |  | 0.98 | 0.80 | 1.20 |  |
| Latvia | 42 | 0.87 | 0.56 | 1.35 |  | 0.93 | 0.60 | 1.45 |  | 0.98 | 0.63 | 1.52 |  |
| **Central Europe** | 763 | **0.81** | **0.73** | **0.90** |  | **0.83** | **0.75** | **0.92** |  | **0.84** | **0.76** | **0.93** |  |
| Poland | 371 | **0.82** | **0.71** | **0.95** |  | 0.87 | 0.75 | 1.00 |  | 0.87 | 0.75 | 1.00 |  |
| Other Central Europe | 144 | 0.87 | 0.69 | 1.11 |  | 0.88 | 0.69 | 1.11 |  | 0.89 | 0.70 | 1.13 |  |
| Hungary | 248 | **0.76** | **0.63** | **0.91** |  | **0.76** | **0.64** | **0.91** |  | **0.78** | **0.65** | **0.93** |  |
| **Africa** | 590 | **0.76** | **0.68** | **0.86** |  | 0.93 | 0.83 | 1.05 |  | 0.93 | 0.83 | 1.05 |  |
| **Northern America** | 224 | **0.58** | **0.48** | **0.70** |  | **0.69** | **0.57** | **0.83** |  | **0.76** | **0.63** | **0.92** |  |
| **Latin America** | 532 | **0.82** | **0.73** | **0.93** |  | 0.95 | 0.84 | 1.08 |  | 0.97 | 0.86 | 1.10 |  |
| Chile | 342 | 0.90 | 0.77 | 1.05 |  | 1.01 | 0.87 | 1.18 |  | 1.02 | 0.87 | 1.19 |  |
| South America | 190 | 0.72 | 0.58 | 0.88 |  | 0.87 | 0.71 | 1.07 |  | 0.90 | 0.73 | 1.11 |  |
| **Asia** | 4169 | **1.26** | **1.20** | **1.31** |  | **1.45** | **1.39** | **1.52** |  | **1.33** | **1.27** | **1.39** |  |
| Turkey | 701 | **1.27** | **1.14** | **1.41** |  | **1.47** | **1.31** | **1.63** |  | **1.34** | **1.20** | **1.50** |  |
| Lebanon | 382 | **1.38** | **1.19** | **1.59** |  | **1.57** | **1.36** | **1.82** |  | **1.45** | **1.25** | **1.68** |  |
| Iran | 891 | **1.20** | **1.09** | **1.32** |  | **1.28** | **1.16** | **1.41** |  | **1.13** | **1.02** | **1.24** |  |
| Iraq | 1281 | **1.62** | **1.50** | **1.76** |  | **2.07** | **1.91** | **2.25** |  | **1.74** | **1.60** | **1.89** |  |
| Other Asia countries | 914 | 0.96 | 0.87 | 1.05 |  | **1.13** | **1.02** | **1.24** |  | **1.14** | **1.04** | **1.25** |  |
| **Russia** | 111 | **0.74** | **0.57** | **0.97** |  | 0.85 | 0.65 | 1.11 |  | 0.86 | 0.66 | 1.13 |  |
| Model 1: adjusted for age and region of residence in Sweden; model 2: adjusted for age, region of residence in Sweden, educational level, and marital status, and neighborhood deprivation; model 3: model 2 + comorbidities | | | | | | | | | | | | | |

| **Supplementary Table 4b. Incidence of hearing impairment in first-generation female immigrants vs Swedish-born women expressed as hazard ratios (HR) with 99% confidence intervals (99% CI)** | | | | | | | | | | | | | |
| --- | --- | --- | --- | --- | --- | --- | --- | --- | --- | --- | --- | --- | --- |
|  |  | Model 1 | | |  | Model 2 | | |  | Model 3 | | |  |
|  | Obs. | HR | 99% CI | |  | HR | 99% CI | |  | HR | 99% CI | |  |
| Sweden | 105260 | 1 |  |  |  | 1 |  |  |  | 1 |  |  |  |
| **Nordic countries** | 6203 | **0.81** | **0.78** | **0.84** |  | **0.90** | **0.86** | **0.93** |  | **0.91** | **0.87** | **0.94** |  |
| Denmark | 692 | **0.72** | **0.64** | **0.80** |  | **0.79** | **0.70** | **0.88** |  | **0.80** | **0.72** | **0.90** |  |
| Finland | 4580 | **0.85** | **0.81** | **0.89** |  | **0.94** | **0.90** | **0.98** |  | **0.94** | **0.90** | **0.99** |  |
| Iceland | 43 | **0.37** | **0.24** | **0.57** |  | **0.47** | **0.31** | **0.73** |  | **0.51** | **0.33** | **0.78** |  |
| Norway | 888 | **0.75** | **0.68** | **0.82** |  | **0.84** | **0.76** | **0.92** |  | **0.85** | **0.77** | **0.94** |  |
| **Southern Europe** | 311 | **0.48** | **0.40** | **0.56** |  | **0.58** | **0.49** | **0.68** |  | **0.62** | **0.52** | **0.72** |  |
| France | 41 | **0.50** | **0.32** | **0.79** |  | **0.59** | **0.38** | **0.92** |  | **0.61** | **0.39** | **0.96** |  |
| Greece | 122 | **0.43** | **0.33** | **0.56** |  | **0.55** | **0.43** | **0.72** |  | **0.60** | **0.46** | **0.77** |  |
| Italy | 63 | **0.43** | **0.30** | **0.62** |  | **0.53** | **0.37** | **0.76** |  | **0.56** | **0.39** | **0.80** |  |
| Spain | 54 | **0.63** | **0.43** | **0.93** |  | 0.75 | 0.51 | 1.11 |  | 0.78 | 0.53 | 1.15 |  |
| Other Southern Europe | 31 | **0.51** | **0.31** | **0.85** |  | **0.59** | **0.36** | **0.99** |  | 0.61 | 0.37 | 1.02 |  |
| **Western Europe** | 1248 | **0.89** | **0.82** | **0.97** |  | 0.94 | 0.86 | 1.02 |  | 0.93 | 0.86 | 1.01 |  |
| The Netherlands | 80 | 0.94 | 0.68 | 1.29 |  | 1.06 | 0.77 | 1.46 |  | 1.10 | 0.80 | 1.51 |  |
| UK and Ireland | 123 | **0.55** | **0.43** | **0.72** |  | **0.65** | **0.50** | **0.84** |  | **0.68** | **0.52** | **0.87** |  |
| Germany | 910 | 1.00 | 0.91 | 1.10 |  | 1.02 | 0.93 | 1.12 |  | 1.00 | 0.91 | 1.10 |  |
| Austria | 94 | 0.78 | 0.58 | 1.04 |  | 0.80 | 0.60 | 1.07 |  | 0.79 | 0.59 | 1.06 |  |
| Other Western Europe | 41 | 0.66 | 0.42 | 1.03 |  | 0.74 | 0.47 | 1.16 |  | 0.76 | 0.48 | 1.18 |  |
| **Eastern Europe** | 1488 | 0.95 | 0.88 | 1.02 |  | **1.13** | **1.05** | **1.22** |  | **1.13** | **1.05** | **1.22** |  |
| Bosnia | 357 | 0.91 | 0.78 | 1.06 |  | **1.24** | **1.07** | **1.45** |  | **1.21** | **1.04** | **1.41** |  |
| Yugoslavia | 859 | 0.95 | 0.86 | 1.05 |  | **1.11** | **1.01** | **1.23** |  | **1.12** | **1.01** | **1.23** |  |
| Croatia | 67 | 1.01 | 0.71 | 1.43 |  | 1.11 | 0.79 | 1.58 |  | 1.12 | 0.79 | 1.59 |  |
| Romania | 113 | 0.90 | 0.68 | 1.17 |  | 0.95 | 0.73 | 1.25 |  | 0.95 | 0.72 | 1.24 |  |
| Bulgaria | 39 | 0.96 | 0.61 | 1.52 |  | 1.11 | 0.70 | 1.75 |  | 1.12 | 0.71 | 1.77 |  |
| Other Eastern Europe | 53 | 1.17 | 0.79 | 1.73 |  | 1.45 | 0.98 | 2.16 |  | 1.47 | 0.99 | 2.19 |  |
| **Baltic countries** | 241 | 0.85 | 0.70 | 1.02 |  | 0.85 | 0.71 | 1.02 |  | 0.85 | 0.71 | 1.03 |  |
| Estonia | 204 | 0.88 | 0.72 | 1.07 |  | 0.88 | 0.72 | 1.07 |  | 0.88 | 0.72 | 1.07 |  |
| Latvia | 37 | 0.70 | 0.44 | 1.12 |  | 0.73 | 0.46 | 1.17 |  | 0.74 | 0.47 | 1.19 |  |
| **Central Europe** | 919 | 0.93 | 0.84 | 1.02 |  | 0.94 | 0.86 | 1.04 |  | 0.94 | 0.85 | 1.03 |  |
| Poland | 559 | 0.90 | 0.80 | 1.02 |  | 0.93 | 0.82 | 1.05 |  | 0.92 | 0.82 | 1.04 |  |
| Other Central Europe | 131 | 0.90 | 0.70 | 1.15 |  | 0.89 | 0.69 | 1.14 |  | 0.88 | 0.69 | 1.13 |  |
| Hungary | 229 | 1.01 | 0.84 | 1.22 |  | 1.01 | 0.84 | 1.22 |  | 1.01 | 0.83 | 1.21 |  |
| **Africa** | 379 | 1.04 | 0.89 | 1.20 |  | **1.32** | **1.14** | **1.53** |  | **1.32** | **1.14** | **1.53** |  |
| **Northern America** | 172 | **0.58** | **0.47** | **0.72** |  | **0.67** | **0.54** | **0.83** |  | **0.68** | **0.55** | **0.85** |  |
| **Latin America** | 498 | 0.95 | 0.84 | 1.08 |  | 1.07 | 0.94 | 1.22 |  | 1.08 | 0.95 | 1.22 |  |
| Chile | 298 | 1.01 | 0.85 | 1.19 |  | 1.11 | 0.94 | 1.31 |  | 1.11 | 0.94 | 1.31 |  |
| South America | 200 | 0.88 | 0.72 | 1.08 |  | 1.02 | 0.83 | 1.25 |  | 1.03 | 0.84 | 1.26 |  |
| **Asia** | 2874 | **1.27** | **1.20** | **1.34** |  | **1.55** | **1.47** | **1.64** |  | **1.53** | **1.44** | **1.62** |  |
| Turkey | 521 | **1.41** | **1.24** | **1.59** |  | **1.72** | **1.52** | **1.95** |  | **1.67** | **1.47** | **1.89** |  |
| Lebanon | 214 | **1.36** | **1.12** | **1.66** |  | **1.70** | **1.40** | **2.07** |  | **1.69** | **1.39** | **2.05** |  |
| Iran | 582 | **1.32** | **1.17** | **1.48** |  | **1.49** | **1.32** | **1.68** |  | **1.42** | **1.26** | **1.60** |  |
| Iraq | 630 | **1.55** | **1.38** | **1.74** |  | **2.14** | **1.90** | **2.40** |  | **2.03** | **1.80** | **2.28** |  |
| Other Asia countries | 927 | 1.03 | 0.94 | 1.14 |  | **1.26** | **1.15** | **1.39** |  | **1.30** | **1.18** | **1.43** |  |
| **Russia** | 177 | 0.84 | 0.68 | 1.04 |  | 0.96 | 0.77 | 1.19 |  | 0.96 | 0.78 | 1.19 |  |
| Model 1: adjusted for age and region of residence in Sweden; model 2: adjusted for age, region of residence in Sweden, educational level, and marital status; model 3: model 2 + neighborhood deprivation. | | | | | | | | | | | | | |

| **Supplementary Table 5. HR of visual impairment in first-generation immigrants, adjusted for duration of stay in Sweden.** | | | | | | | | |
| --- | --- | --- | --- | --- | --- | --- | --- | --- |
|  | Men | | |  | Women | | | |
|  | HR* | 99% CI | |  | HR* | 99% CI | |  |
| Sweden | 1.00 |  |  |  | 1.00 |  |  |  |
| **All foreign-born** | **0.84** | **0.81** | **0.87** |  | **0.88** | **0.85** | **0.92** |  |
| **Nordic countries** | **0.77** | **0.73** | **0.80** |  | **0.84** | **0.80** | **0.88** |  |
| **Southern Europe** | **0.63** | **0.56** | **0.70** |  | **0.58** | **0.49** | **0.69** |  |
| **Western Europe** | **0.78** | **0.72** | **0.85** |  | **0.88** | **0.81** | **0.96** |  |
| **Eastern Europe** | 0.97 | 0.91 | 1.04 |  | 0.99 | 0.91 | 1.07 |  |
| **Baltic countries** | 0.95 | 0.79 | 1.14 |  | 0.84 | 0.69 | 1.00 |  |
| **Central Europe** | **0.77** | **0.69** | **0.85** |  | **0.82** | **0.74** | **0.90** |  |
| **Africa** | **0.84** | **0.74** | **0.95** |  | 1.14 | 0.98 | 1.33 |  |
| **Northern America** | **0.70** | **0.58** | **0.85** |  | **0.69** | **0.55** | **0.86** |  |
| **Latin America** | **0.87** | **0.77** | **0.99** |  | 0.92 | 0.80 | 1.05 |  |
| **Asia** | **1.19** | **1.12** | **1.26** |  | **1.26** | **1.18** | **1.34** |  |
| **Russia** | 0.78 | 0.60 | 1.03 |  | 0.87 | 0.70 | 1.08 |  |
|  |  |  |  |  |  |  |  |  |

*Fully adjusted, i.e. adjusted for age, region of residence in Sweden, educational level, and marital status, neighborhood deprivation, and comorbidities

**Supplementary Table 6a. The numbers and percentages of different diagnoses (with ICD-10 codes) for men in the study.**

| **Number OF CASE IN MEN** | | | | | |
| --- | --- | --- | --- | --- | --- |
| **ICD-CODES** | **Frequency** | **Percent** | **Cumulative Frequency** | **Cumulative Percent** |  |
| Noise-induced hearing loss (H83.3) | 4608 | 3.71 | 4608 | 3.71 |  |
| Conductive hearing loss, bilateral (H90.0) | 694 | 0.56 | 5302 | 4.26 |  |
| Conductive hearing loss, unilateral with unrestricted hearing on the contralateral side (H.90.1) | 2673 | 2.15 | 7975 | 6.41 |  |
| Conductive hearing loss, unspecified  (H90.2) | 2142 | 1.72 | 10117 | 8.14 |  |
| Sensorineural hearing loss, bilateral (H90.3) | 48351 | 38.88 | 58468 | 47.02 |  |
| Sensorineural hearing loss, unilateral with unrestricted hearing on the contralateral side  (H90.4) | 7983 | 6.42 | 66451 | 53.44 |  |
| Sensorineural hearing loss, unspecified  (H90.5) | 31027 | 24.95 | 97478 | 78.39 |  |
| Mixed conductive and sensorineural hearing loss, bilateral (H90.6) | 2022 | 1.63 | 99500 | 80.02 |  |
| Mixed conductive and sensorineural hearing (H90.7) | 1063 | 0.85 | 100563 | 80.87 |  |
| Mixed conductive and sensorineural hearing loss, unspecified  (H90.8) | 3758 | 3.02 | 104321 | 83.89 |  |
| Ototoxic hearing loss  (H91.0) | 73 | 0.06 | 104394 | 83.95 |  |
| Presbycusis  (H91.1) | 7516 | 6.04 | 111910 | 90.00 |  |
| Sudden idiopathic hearing loss  (H91.2) | 4745 | 3.82 | 116655 | 93.81 |  |
| Deaf mutism, not elsewhere classified  (H91.3) | 156 | 0.13 | 116811 | 93.94 |  |
| Other specified hearing loss  (H91.8) | 554 | 0.45 | 117365 | 94.38 |  |
| Hearing loss, unspecified  (H91.9) | 6984 | 5.62 | 124349 | 100.00 |  |

**Supplementary Table 6b. The numbers and percentages of different diagnoses (with ICD-10 codes) for women in the study.**

| Number OF CASES IN WOMEN | | | | | |
| --- | --- | --- | --- | --- | --- |
| **ICD-CODES** | **Frequency** | **Percent** | **Cumulative Frequency** | **Cumulative Percent** |  |
| Noise-induced hearing loss (H83.3) | 452 | 0.38 | 452 | 0.38 |  |
| Conductive hearing loss, bilateral (H90.0) | 925 | 0.77 | 1377 | 1.15 |  |
| Conductive hearing loss, unilateral with unrestricted hearing on the contralateral side (H.90.1) | 3656 | 3.05 | 5033 | 4.20 |  |
| Conductive hearing loss, unspecified  (H90.2) | 2936 | 2.45 | 7969 | 6.65 |  |
| Sensorineural hearing loss, bilateral (H90.3) | 43825 | 36.58 | 51794 | 43.23 |  |
| Sensorineural hearing loss, unilateral with unrestricted hearing on the contralateral side  (H90.4) | 8681 | 7.24 | 60475 | 50.47 |  |
| Sensorineural hearing loss, unspecified  (H90.5) | 28594 | 23.86 | 89069 | 74.33 |  |
| Mixed conductive and sensorineural hearing loss, bilateral (H90.6) | 2405 | 2.01 | 91474 | 76.34 |  |
| Mixed conductive and sensorineural hearing (H90.7) | 1413 | 1.18 | 92887 | 77.52 |  |
| Mixed conductive and sensorineural hearing loss, unspecified  (H90.8) | 4262 | 3.56 | 97149 | 81.08 |  |
| Ototoxic hearing loss  (H91.0) | 97 | 0.08 | 97246 | 81.16 |  |
| Presbycusis  (H91.1) | 9316 | 7.77 | 106562 | 88.93 |  |
| Sudden idiopathic hearing loss  (H91.2) | 4210 | 3.51 | 110772 | 92.45 |  |
| Deaf mutism, not elsewhere classified  (H91.3) | 155 | 0.13 | 110927 | 92.58 |  |
| Other specified hearing loss  (H91.8) | 666 | 0.56 | 111593 | 93.13 |  |
| Hearing loss, unspecified  (H91.9) | 8228 | 6.83 | 119822 | 100.00 |  |
